# 245. Risk Factors Associated with Open Fracture Complications Following Antibiotic Prophylaxis

**DOI:** 10.1093/ofid/ofab466.447

**Published:** 2021-12-04

**Authors:** Elizabeth Cusack, Kaylee Maynard, Ted Louie, John Gorczyca, Courtney M Jones, Nicole M Acquisto, Stephanie Shulder

**Affiliations:** 1 University of Rochester Medical Center- Strong Memorial Hospital, Rochester, New York; 2 University of Rochester Medical Center, Rochester, New York; 3 University of Rochester, Rochester, New York; 4 University of Rochester, School of Medicine, Rochester, New York

## Abstract

**Background:**

Surgical site infection is concerning after an open fracture. The Eastern Association for the Surgery of Trauma guidelines provide antibiotic selection and duration recommendations based on open fracture type. Risk factors for open fracture complications (e.g. infection, acute kidney injury [AKI], multi-drug resistant organisms [MDRO], or *Clostridioides* infection [*C. difficile*]) and overall guideline adherence are unclear at our institution.

**Methods:**

This was a single center, retrospective study of adult patients with an open fracture who received antibiotic prophylaxis and were admitted for at least 24 hours between March 2011 and October 2020. Patients were excluded if open fracture was due to gun-shot wound, had a history of renal replacement therapy, MDRO, or *C. difficile* infection, were an outside hospital transfer, received antibiotics for another indication, or had a delayed presentation. The primary outcome was to identify risk factors for infection and secondary outcomes to identify risk factors for AKI, MDRO, *C. difficile* infection, and to evaluate guideline adherence. Patient demographics including injury details and management, microbiologic cultures, and antibiotic information were collected. Data were analyzed by univariate analysis, as appropriate, and logistic regression.

**Results:**

A total of 401 patients met study criteria; median age 46 years, 62% male, and 77% white. Fracture classifications were similar: 30% type I, 39% type II, and 30% type III. Infection occurred in 18% of patients, AKI in 18%, MDRO in 3%, and no patients developed *C. difficile*. Of those with culture-positive infection, 51% grew gram-positive organisms. In bivariate analysis, fracture classification (p=0.023), medical fracture management (p=0.034), and antibiotic choice (p=0.004) were associated with infection. The only independent risk factor associated with AKI was receiving a nephrotoxic medication (p=0.012). Eighty-one percent received guideline adherent antibiotics and of those that received too narrow antibiotics, 36% developed an infection (p=0.004).

**Conclusion:**

Appropriate fracture classification and antibiotic choice is crucial to reduce infection following open fracture. Reducing concomitant exposure to nephrotoxic agents may reduce the risk of AKI.

**Disclosures:**

**All Authors**: No reported disclosures

